# Induction of G2/M Cell Cycle Arrest and Apoptosis by Genistein in Human Bladder Cancer T24 Cells through Inhibition of the ROS-Dependent PI3k/Akt Signal Transduction Pathway

**DOI:** 10.3390/antiox8090327

**Published:** 2019-08-21

**Authors:** Cheol Park, Hee-Jae Cha, Hyesook Lee, Hyun Hwang-Bo, Seon Yeong Ji, Min Yeong Kim, Su Hyun Hong, Jin-Woo Jeong, Min Ho Han, Sung Hyun Choi, Cheng-Yun Jin, Gi-Young Kim, Yung Hyun Choi

**Affiliations:** 1 Department of Molecular Biology, College of Natural Sciences, Dong-eui University, Busan 47340, Korea; 2 Department of Parasitology and Genetics, Kosin University College of Medicine, Busan 49267, Korea; 3 Anti-Aging Research Center, Dong-eui University, Busan 47340, Korea; 4 Department of Biochemistry, Dong-eui University College of Korean Medicine, Busan 47227, Korea; 5 Freshwater Bioresources Utilization Bureau, Nakdonggang National Institute of Biological Resources, Sangju 37242, Korea; 6 National Marine Biodiversity Institute of Korea, Seocheon 33662, Korea; 7 Department of System Management, Korea Lift College, Geochang 50141, Korea; 8 School of Pharmaceutical Sciences, Collaborative Innovation Center of New Drug Research and Safety Evaluation, Zhengzhou University, Henan 450001, China; 9 Department of Marine Life Sciences, Jeju National University, Jeju 63243, Korea

**Keywords:** genistein, G2/M arrest, apoptosis, ROS, PI3K/Akt

## Abstract

We examined the anti-cancer effect of genistein, a soy-derived isoflavone, in human bladder transitional cell carcinoma T24 cells. According to our data, genistein induced G2/M phase arrest of the cell cycle and apoptosis. Genistein down-regulated the levels of cyclin A and cyclin B1, but up-regulated the levels of p21WAF1/CIP1, cyclin-dependent kinase (Cdk) inhibitor, that was complexed with Cdc2 and Cdk2. Furthermore, genistein induced the activation of caspases (caspase-3, -8 and -9), and cleavage of poly (ADP-ribose) polymerase cleavage. However, genistein-induced apoptosis was significantly inhibited by a pan-caspase inhibitor, indicating that the induction of apoptosis by genestein was caspase-dependent. In addition, genistein increased the cytosolic release of cytochrome *c* by increasing the Bax/Bcl-2 ratio and destroying mitochondria integrity. Moreover, genistein inactivated the phosphoinositide 3-kinase (PI3K)/Akt signaling pathway, while LY294002, a PI3K/Akt inhibitor, increased the apoptosis-inducing effect of genistein. Genistein further increased the accumulation of reactive oxygen species (ROS), which was significantly suppressed by N-acetyl cysteine (NAC), a ROS scavenger, and in particular, NAC prevented genistein-mediated inactivation of PI3K/Akt signaling, G2/M arrest and apoptosis. Therefore, the present results indicated that genistein promoted apoptosis induction in human bladder cancer T24 cells, which was associated with G2/M phase cell cycle arrest via regulation of ROS-dependent PI3K/Akt signaling pathway.

## 1. Introduction

Based on an understanding of the signaling mechanisms that regulate the growth of tumor cells over the past decade, effective therapies have been developed for the treatment of cancer patients. However, although various side effects, including limited efficacy and drug resistance, need to be solved, chemotherapy is still the main approach to cancer therapy [1,2,3,4]. Therefore, in order to develop safer and effective therapies that can overcome these problems, there is a growing interest in natural products that can block the proliferation of cancer cells without affecting normal cells [5,6,7,8].

Genistein is a type of isoflavonoids found mainly in soybean products and first identified as an inhibitor of tyrosine protein kinases [9]. Although a number of beneficial actions of genistein are known, studies on the anti-cancer activity have been most extensively carried out [10,11,12]. Although genistein induced cell cycle arrest at G1 and/or S phase in certain cancer cell lines, this isoflavonoid is known to inhibit cancer cell growth through G2/M inhibition in most cancer cells under conditions that are not toxic to normal cells [13,14]. In addition, the excessive production of reactive oxygen species (ROS) in some types of tumor cells plays a critical role in the induction of apoptosis [13,14,15,16,17]. Moreover, the anti-cancer effects of genistein involve the disturbance of various cell signaling pathways. For example, genistein-induced apoptosis in several human cancer cells, including ovarian, lung, colon and breast cancer cells, was accompanied by inactivation of the phosphoinositide 3-kinase (PI3K)/Akt signal transduction pathway [16,18,19]. Although genistein-induced cell cycle arrest and apoptosis in human leukemia cells was accompanied by the production of ROS and inactivation of the PI3K/Akt signaling [20], the underlying mechanism of ROS in genistein-mediated inactivation of PI3K/Akt signaling pathway is still not well known. Furthermore, the possibility of genistein on growth inhibitory activity in bladder cancer cells has been proposed [21,22], no detailed molecular mechanism supporting its effect has been reported. Therefore, here, we investigated the effect of genistein on the induction of cell cycle arrest and apoptosis, and investigated whether its effect was associated with ROS generation and PI3K/Akt signaling pathway inactivation in human urinary bladder transitional cell carcinoma T24 cells.

## 2. Materials and Methods

### 2.1. Cell Culture

The T24 cell line was purchased from the American Type Culture Collection (ATCC, Manassas, MD, USA) and grown in RPMI 1640 medium containing 10% heat-inactivated fetal bovine serum (FBS), and 1% penicillin and streptomycin (WelGENE Inc., Daegu, Korea). Normal non carcinoma cell lines, including myoblast C2C12 cells and lung fibroblast V79-4 cells were also obtained from the ATCC. C2C12 and V79-4 cells were maintained in Dulbecco’s Modified Eagle’s Medium (DMEM, WelGENE Inc.) supplemented with 10% FBS, and 1% penicillin and streptomycin. All cell lines were grown at 37 °C in 5% CO_2_ humidified incubator. Genistein (Sigma-Aldrich Chemical Co., St. Louis, MO, USA) was dissolved in dimethyl sulfoxide (DMSO, Sigma-Aldrich Chemical Co.) and then diluted to the appropriate concentration using culture medium before treatment to the cells.

### 2.2. Cell Viability

The cell viability was examined using 3-(4,5-dimethyl-2-thiazolyl)-2,5-diphenyltetra-zolium bromide (MTT) assay according to the previous method [23]. In brief, 1 × 10^4^ cells per well were plated in 96-well plates. After 24 h, the cells were treated with the desired concentrations of genistein with or without *N*-Benzyloxycarbonyl-Val-Ala-Asp-fluoromethylketone (z-VAD-fmk, Calbiochem, San Diego, CA, USA), N-acetyl-L-cysteine (NAC, Invitrogen, Waltham, MA, USA) or LY294002 (Cell Signaling Technology, Inc., Danvers, MA, USA). After 48 h, the medium was changed with fresh medium containing 50 μg/mL MTT solution (Invitrogen). After 2 h, the medium was removed and added 100 μL of DMSO. Absorbance at 540 nm was measured using a microplate reader (Molecular Device Co., Sunnyvale, CA, USA). The morphological changes of cells following genistein treatment were observed and visualized by a phase-contrast microscope (Carl Zeiss, Oberkochen, Germany).

### 2.3. Flow Cytometric Analysis for Apoptosis, Mitochondrial Membrane Potential (MMP, ΔΨm) and ROS Generation

The cells were fixed in 70% ethanol in phosphate-buffered saline (PBS) for 30 min. After staining with 40 μg/mL propidium iodide (PI, Sigma-Aldrich Chemical Co.) for 30 min, the phase distribution of the cell cycle was determined by using a flow cytometer (BD Biosciences, San Jose, CA, USA) as described previously [24]. To determine and quantify the apoptotic cells by a flow cytometer, the Annexin V-fluorescein isothiocyanate (FITC) staining kit (BD Biosciences) was used, according to the manufacturer’s instruction. The levels of MMP and ROS generation were measured using 5,5′,6,6′-tetrachloro-1,1′,3,3′-tetraethyl-imidacarbocyanine iodide (JC-1; Sigma-Aldrich Chemical Co.) and 5,6-carboxy-2′,7′-dichlorodihydrofluorescein diacetate (DCF-DA, Invitrogen) staining, respectively, by the manufacturer’s recommended protocol.

### 2.4. Observation of Apoptotic Cells

Nuclear morphology changes for assessing apoptosis of cells cultured under various conditions were determined by 4′,6′-diamidino-2-phenylindole (DAPI, Sigma-Aldrich Chemical Co.) staining as according to previously described [24].

### 2.5. Reverse Transcriptase-Polymerase Chain Reaction (RT-PCR)

After treatment, both adherent and floating cells were collected, and total RNA was extracted using TRIzol reagent (Invitrogen) by following the manufacturer’s protocol. After quantifying the RNA concentration, target genes were amplified using AccuPower^®^ PCR PreMix (Bioneer, Daejeon, Korea), as described previously [25]. Glyceraldehyde 3-phosphate dehydrogenase (GAPDH) was used as a control for RNA expression.

### 2.6. Immunoprecipitation and Western Blot Analysis

Co-immunoprecipitation and immunoblotting analysis were performed as described previously [24,26] for the investigation of protein expression. Specific primary and horseradish peroxidase-conjugated secondary antibodies were obtained from Cell Signaling Technology (Danvers, MA, USA) and Santa Cruz Biotechnology, Inc. (Santa Cruz, CA, USA). The blots were visualized using by enhanced chemiluminescence (ECL) kit (GE Healthcare Life Sciences, Little Chalfont, UK) and Image system (Vilber Lourmat, Torcy, France). Actin was used as housekeeper and loading control.

### 2.7. Immunofluorescence Staining

The cells were cultured on coverslips, and then cells were stimulated with 160 μM of genistein for 24 h and 48 h. After fixing with 4% paraformaldehyde in PBS for 15 min, the cells were treated with 0.2% Triton X-100 in PBS for 15 min and blocked with 5% bovine serum albumin (Sigma-Aldrich Chemical Co.) for 10 min. Cells were stained overnight at 4 °C with rabbit antibody against anti-phospho-histone H3 (Ser 10, Santa Cruz Biotechnology, Inc.). The cells were then stained with a fluorescein-conjugated anti-rabbit IgG in the dark at 37 °C for 1 h. After staining the nuclei using DAPI, the cells were mounted on slides and analyzed by a fluorescence microscope (Carl Zeiss).

### 2.8. Assessment of Caspase Activity

The activities of caspases (caspase-3, -8 and -9) were measured by colorimetric assays as previously described [24]. The absorbance was determined using a microplate reader at 405 nm.

### 2.9. Statistical Analysis

Data were represented as mean ± standard deviation (SD) from at least three independent experiments. Statistical analysis was analyzed using GraphPad Prism software (GraphPad Software, Inc., La Jolla, CA, USA), and significance tests were performed using one-way ANOVA with Tukey’s test. Statistically differences were expressed * *p* < 0.05, ** *p* < 0.001 and *** *p* < 0.0001 compared to control; ^#^*p* < 0.05, ^##^ *p* < 0.001 and ^###^ *p* < 0.0001 compared to genistein-treated cells.

## 3. Results

### 3.1. Inhibition of T24 Cell Viability by Genistein

**Figure 1:**
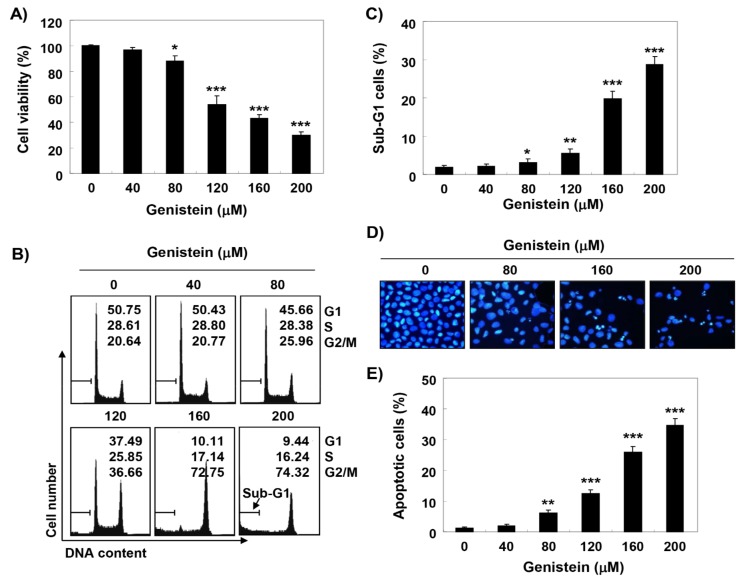
Induction of cell cycle arrest at the G2/M phase and apoptosis by genistein in T24 cells (**A**) After treatment with genistein for 48 h, the cell viability was investigated as described in the Materials and Methods. Each bar indicated the mean ± standard deviation (SD, * *p* < 0.05 and *** *p* < 0.0001 compared to control). (**B**) The effects of genistein on cell cycle distribution. The percentages of G1, S and G2/M population were plotted in the histograms. (**C**) The apoptotic sub-G1 fraction population was expressed as a percentage relative to total cells. (**D**) The nuclear morphology was examined using 4′,6′-diamidino-2-phenylindole (DAPI) staining. (**E**) The frequencies of apoptotic cells were revealed as a percentage of Annexin V-positive cells (** *p* < 0.001 and *** *p* < 0.0001 compared to control).

To determine the cytotoxic effect of genistein on the growth of T24 cells for 48 h, cell viability was assessed by an MTT assay. Figure 1A shows that genistein significantly reduced T24 cell viability in a concentration of over 80 μM in a dose-dependent manner. Under the phase-contrast microscope, the morphology of genistein-stimulated cells indicated irregular shapes of the cell, a decrease of the cell population, and an increase of detached cell (data not shown). We also compared the cell viability after treatment with genistein in normal cells, including C2C12 and V79-4 cells (Supplementary Figure S1A).

### 3.2. G2/M Arrest and Apoptosis Induction by Genistein in T24 Cells

To explore the mechanism for the genistein-induced anti-proliferative effect in T24 cells, the cell cycle distribution profile was assessed. Figure 1B showed that genistein concentration-dependently increased the frequency of arrested cells at G2/M phase, and simultaneously decreased the cells population in G1 and S phases. In the meanwhile, a significant increase of the cells at apoptotic sub-G1 phase with increasing genistein treatment concentration was observed (Figure 1C). Especially, treatment of T24 cells with 160 μM of genistein for 48 h led to a two-fold higher number of cells in the G2/M phase as compared for 24 h (Supplementary Figure S2). In addition, DAPI staining that genistein increased the frequency of cells containing chromatin condensation, apoptotic body formation (Figure 1D). Furthermore, the populations of annexin V^+^ cells were markedly increased, as compared to the control, indicating that genistein-mediated cell cycle arrest at the G2/M phase was related to the induction of apoptosis (Figure 1E). While genistein did not affect the cell cycle arrest in normal cell lines, including C2C12 and V79-4 cells (Supplementary Figure S1C).

### 3.3. Effects of Genistein on the Expression of Cell Cycle Regulatory Genes in T24 Cells

**Figure 2:**
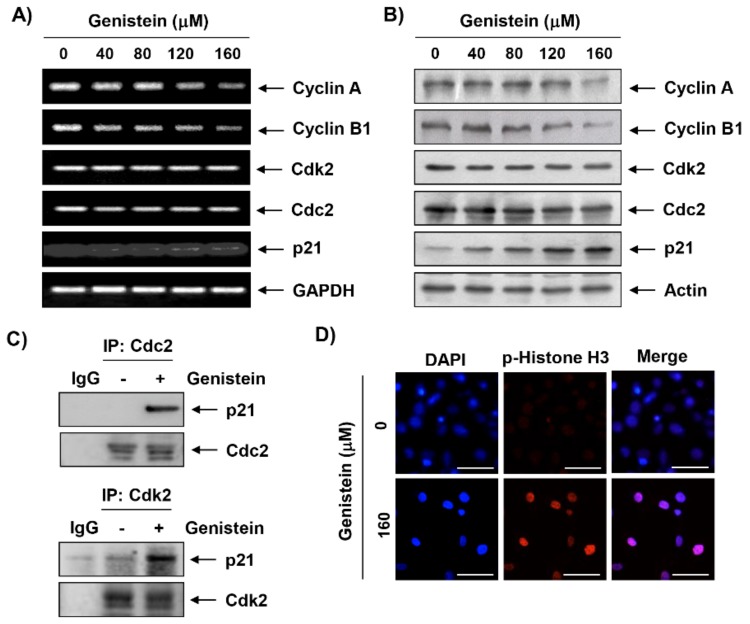
Effects of genistein on the expression of cell cycle regulatory genes in T24 cells. (**A**) The cells were treated with different concentration of genistein for 48 h, and then mRNA levels were determined using a reverse transcriptase-polymerase chain reaction (RT-PCR) assay. (**B**) Protein levels of cell cycle regulatory genes were measured by Western blot analysis. (**C**) Co-immunoprecipitation (IP) assay indicated that genistein-induced p21 interacted with Cdc2 and Cdk2. IgG serves as a negative control of IP. (**D**) Cells were stained with 5hosphor-histone H3 (Ser 10) antibody (red) and DAPI (nuclear stain; blue), then the cells were visualized using a fluorescence microscope (scale bar; 50 μm).

To investigate the mechanism of the genistein-induced cell cycle arrest in T24 cells, the levels of G2/M phase-associated genes were analyzed. The RT-PCR and immunoblotting results indicated that genistein decreased the expression of cyclin A and B1 mRNA and protein in a concentration-dependent manner, while the expression of CdK2 and Cdc2 remained at the control level (Figure 2A,B). Nevertheless, the expression of a Cdk inhibitor p21WAF1/CIP1 was markedly up-regulated by genistein at the mRNA and protein levels in response to genistein exposure. We next performed a co-immunoprecipitation assay to identify the role of genistein-induced p21. As shown in Figure 2C, we found that up-regulated p21 by genistein was apparently combined with Cdc2 and Cdk2. To confirm whether the genistein induces G2 or M arrest, we evaluated the effect of genistein on the expression of phospho-histone H3 (Ser 10), a hallmark of mitosis that plays a pivotal role in the regulation of apoptosis and mitotic catastrophe [25]. Our data showed that genistein treatment markedly increased the expression of 5hosphor-histone H3 for both 24 h (Supplementary Figure S2) and 48 h treatment groups (Figure 2D). Overall, these data support that genistein induced G2/M arrest, including mitotic catastrophe, in T24 cells.

### 3.4. Activation of Caspases by Genistein in T24 Cells

**Figure 3:**
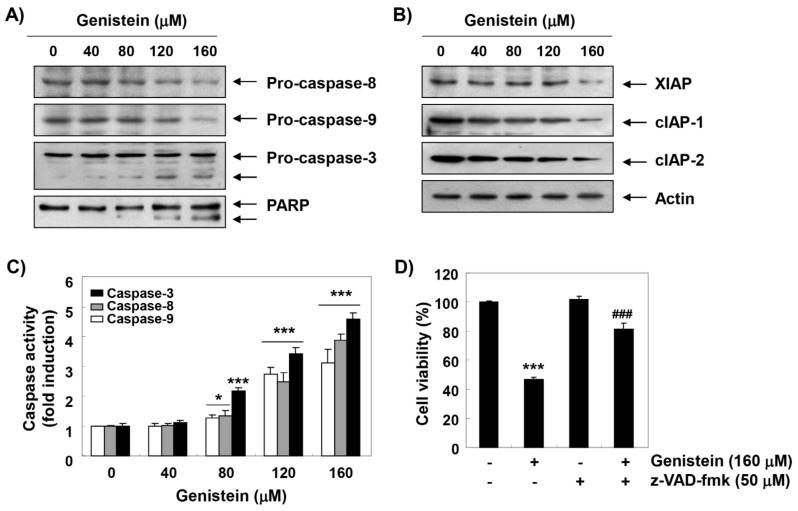
Activation of caspases and cleavage of poly (ADP-ribose) polymerase (PARP) by genistein in T24 cells. (**A**,**B**) The cells were stimulated with different concentrations of genistein. After 48 h, the expression of caspases, poly (ADP-ribose) polymerase (PARP), and members of the inhibitor of apoptosis protein (IAP) family were determined. (**C**) The activities of caspases were examined using colorimetric caspase assay kits. (**D**) The cells were pre-treated with 50 μM *N*-benzyloxycarbonyl-Val-Ala-Asp-fluoromethylketone (z-VAD-fmk), a pan-caspase inhibitor, for 1 h and then cultured in the presence or absence of 160 μM genistein for 48 h, and then, the cell viability was assessed (*** *p* < 0.0001 compared to control; ^###^ *p* < 0.0001 compared to genistein-treated cells).

We examined whether genistein stimulated the caspase pathway during genistein-induced apoptosis. Our results indicated that genistein suppressed the expression of pro-caspases (caspase-8, -9, and -3), and increased their enzymatic activity in a concentration-dependent manner (Figure 3A,C), which was associated with the suppression of members of the inhibitor of apoptosis protein (IAP) family, including XIAP, cIAP-1 and cIAP-2 (Figure 3B). Genistein also induced the degradation of poly (ADP-ribose) polymerase (PARP) (Figure 3A). However, pretreatment with Z-VAD-fmk, a pan-caspase inhibitor, significantly protected the inhibition of cell proliferation by genistein (Figure 3D).

### 3.5. Induction of Mitochondrial Dysfunction by Genistein in T24 Cells

**Figure 4:**
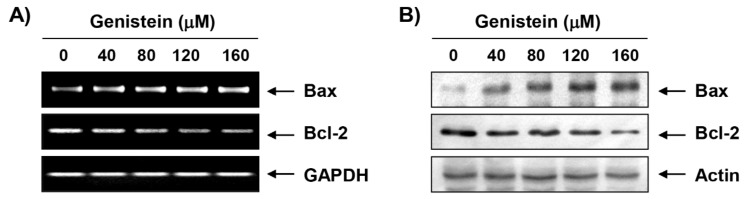

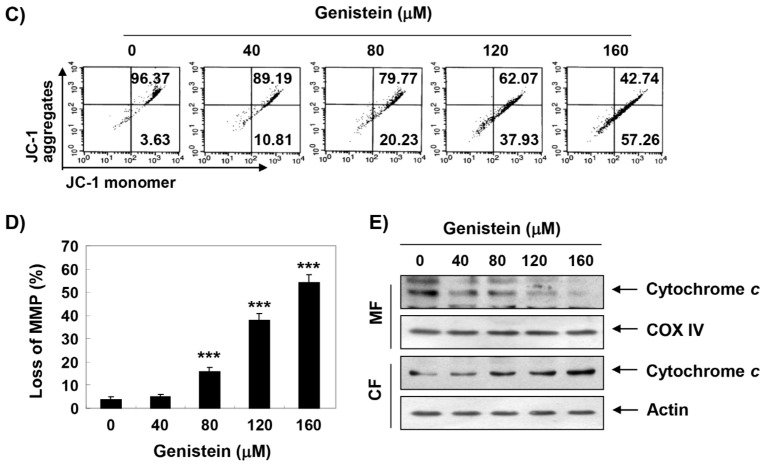
Induction of mitochondrial dysfunction by genistein in T24 cells. The cells were cultured for 48 h in media containing different concentrations of genistein. (**A**,**B**) The levels of mRNA and protein of Bcl-2 family members were examined by RT-PCR and Western blot analysis. (**C**,**D**) The effect of genistein on mitochondrial membrane potential (MMP) was analyzed by a flow cytometer (*** *p* < 0.0001, when compared to control). (**E**) Cytoplasmic and mitochondrial proteins were isolated for analysis of cytochrome *c* expression, and Western blot analysis was performed. Analysis of cytochrome oxidase subunit VI (COX VI) and actin expression was performed to confirm the protein loading of each fraction extract.

To observe the expression of Bcl-2 family members by genistein, RT-PCR and Western blot analysis were performed. Figure 4A,B showed that genistein not only up-regulated the expression of Bax, but also down-regulated the expression of Bcl-2 (Figure 4A,B). In addition, we found that genistein increased the loss of MMP (Figure 4C,D) and the expression of cytochrome *c* in genistein-treated T24 cells was higher than that of mitochondria in cytoplasm (Figure 4E).

### 3.6. Inactivation of PI3K/Akt Signaling Pathway by Genistein in T24 Cells

**Figure 5:**
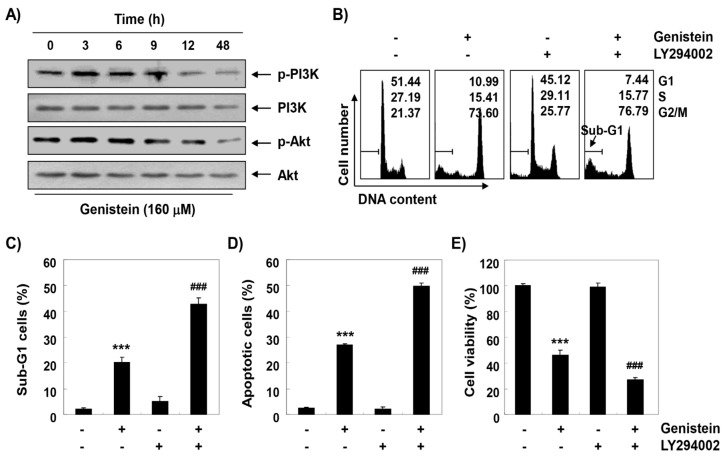
Blocking of phosphoinositide 3-kinase (PI3K)/Akt signaling pathway activity by genistein in T24 cells. (**A**) After treatment with 160 μM genistein for the indicated times, the levels of PI3K and Akt protein were evaluated by Western blot analysis. (**B**–**E**) The cells were pre-treated with 10 μM LY294002 for 1 h and then treated with 160 μM genistein for 48 h. (**B**) After flow cytometry analysis, the percentages of G1, S and G2/M phase population were plotted in the histograms. (**C**,**D**) The percentages of sub-G1 and Annexin V-positive cells were determined. (**E**) The effect of LY294002 on the genistein-induced cell viability reduction was determined by an MTT assay (*** *p* < 0.0001, when compared to control; ### *p* < 0.001, when compared to genistein-treated cells).

To evaluate the effect of genistein on the PI3K/Akt signal transduction pathway in T24 cells, the level of PI3K and its downstream component, Akt, was assessed. Figure 5A shows that when cells were exposed to genistein, the expressions of phosphorylated (p)-PI3K and p-Akt were gradually decreased with increasing time of genistein treatment, while total levels of PI3K and Akt protein remained unchanged during genistein treatment, indicating that genistein was able to block the activation of the PI3K/Akt pathway. To further confirm the role of the PI3K/Akt pathway in genistein-mediated G2/M arrest apoptosis, cells were treated with LY294002, a selective inhibitor of PI3K, in the presence or absence of genistein. The results obtained from flow cytometric analysis showed that induction of apoptosis was significantly increased in cells treated with LY294002 and genistein together compared with cells treated genistein alone, with no significant difference in cell cycle distribution (Figure 5B–D). In addition, after combination treatment with LY294002 and genistein, the reduction of cell viability by genistein was further enhanced (Figure 5E).

### 3.7. Induction of Mitochondrial Dysfunction by Genistein through Stimulating ROS Generation in T24 Cells

**Figure 6:**
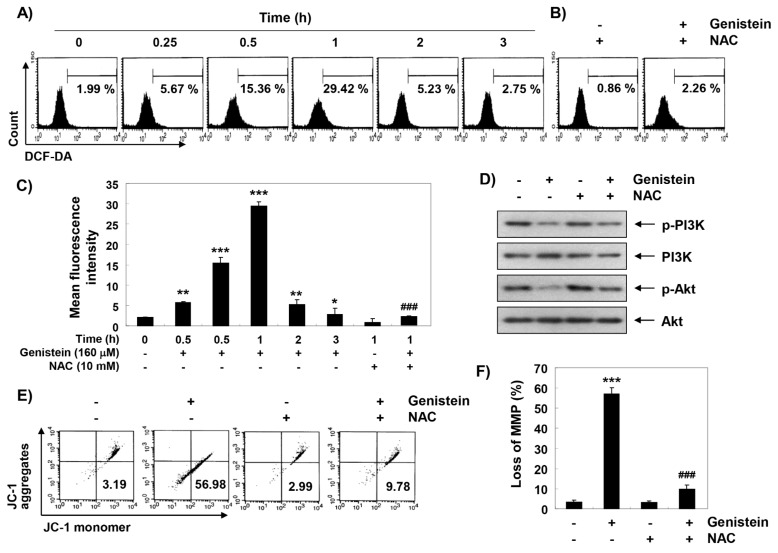
Generation of reactive oxygen species (ROS) and mitochondria dysfunction by genistein in T24 cells. (**A**–**C**) The cells were stimulated with 160 μM genistein for the indicated times or incubated with or without 160 μM genistein for 1 h before treatment with 10 mM N-acetyl-L-cysteine (NAC) for 1 h. Intracellular ROS generation was assessed by a flow cytometer. (**C**) Each bar expressed as the fluorescence intensity of DCF-DA dye (* *p* < 0.05, ** *p* < 0.001 and *** *p* < 0.0001 compared to control; ^###^ *p* < 0.0001 compared to genistein-treated cells). (**D**–**F**) The cells were stimulated with 160 μM genistein for 48 h with or without NAC. (**D**) The expression of PI3K and Akt proteins was evaluated by immunoblotting using the indicated antibodies. (**E**,**F**) Effect of NAC on the genistein-induced loss of MMP was evaluated (mean ± SD of triplicate determinations, *** *p* < 0.0001, when compared to control; ### *p* < 0.001, when compared to genistein-treated cells).

To measure the involvement of ROS on the genistein-mediated inactivation of the PI3K/Akt signaling pathway, flow cytometry analysis was performed using DCF-DA dye. Our data indicated that intracellular ROS production was the greatest increase within 1 h by genistein treatment, whereas it was markedly decreased by NAC treatment, a ROS scavenger (Figure 6A–C). Additionally, genistein-induced decreasing of phosphorylation of PI3K and Akt were recovered to the control levels under the ROS generation was artificially blocked by NAC treatment (Figure 6D). Moreover, the presence of NAC significantly abolished the loss of MMP by genistein (Figure 6E,F).

### 3.8. ROS Plays as a Critical Regulator of Growth Inhibition and Apoptosis by Genistein in T24 Cells

**Figure 7:**
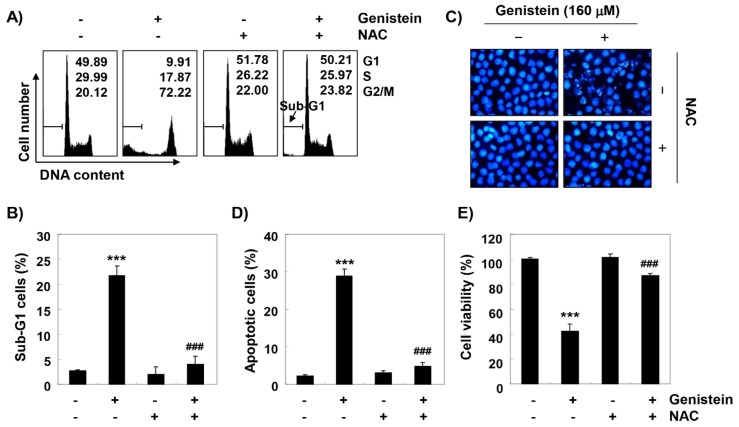
Roles of ROS on the genistein-induced G2/M arrest and apoptotic cell death. T24 cells were cultured in medium containing 160 μM genistein for 48 h or treated with 10 mM NAC for 1 h and then stimulated with genistein for 48 h. (**A**,**B**,**D**) The effect of NAC on the genistein-induced cell cycle arrest and apoptosis were investigated using flow cytometry analysis. (**C**) The nuclear morphology was observed using DAPI staining. (**E**) The effect of NAC on the genistein-induced cytotoxicity was evaluated by an MTT assay (*** *p* < 0.0001 compared to control; ^###^ *p* < 0.0001 compared to genistein-treated cells).

To investigate the role of ROS in genistein-mediated G2/M arrest and apoptosis induction, we further performed the analysis of cell cycle distribution. As indicated in Figure 7A, blocking of ROS generation by NAC treatment reinstated genistein-mediated G2/M cell cycle arrest, which was related to the decline of the cell population at sub-G1 (Figure 7B). Consistent with these results, the increased apoptosis by genistein treatment was largely restored by blocking ROS production (Figure 7C,D). Moreover, inhibiting ROS generation significantly abolished the decreased cell viability by genistein (Figure 7E), demonstrating that ROS was shown to be necessary for the contribution of cell cycle arrest at the G2/M phase and apoptosis by genistein in T24 cells.

## 4. Discussion

Numerous studies have been reported that some anti-cancer agents stimulated cell cycle arrest checkpoint and thereby inducing apoptotic cell death. In particular, the uncontrolled cell cycle is a hallmark of tumor cells, and it is contributed to the progression and development of cancer [5,6]. Our result showed that genistein suppressed cell proliferation that associated with the induction of cell cycle arrest in G2/M phase, similar to the results of previous finding using various human cancer cell lines [13,14,26,27,28,29,30,31,32]. The central machines that drive cell cycle progression are regulated by Cdks that modulated by interactions with cell cycle-specific cyclins and Cdk inhibitors. During the Gl to S phase, cyclin D complex with Cdk4 and Cdk6. Meanwhile, cyclin A/Cdc2 and Cdk2 complex regulate S and G2 phase, and induction of G2/M transition and processes during mitosis are achieved through cyclin B/Cdc2 complex [20,33]. Similar to the results in various cancer cells [26,27,28,30,34], our result demonstrated that genistein decreased the expression of cyclin A and B1, whereas the expression of CdK2 and Cdc2 remained at the control level. In particular, the expression of p21 was markedly up-regulated by genistein. P21 is an important member of the Cip/Kip family of Cdk inhibitor, which also induced tumor suppressor both p53-dependent and p53-independent cell cycle arrest in various cancer type [35,36]. Up-regulated p21 interacted with Cdks to suppress cell cycle progression by inhibiting their kinase activity [35,37]. In the present study, we found that up-regulated p21 by genistein was apparently combined with Cdc2 and Cdk2, which may have involved the inhibition of their kinase activity and finally leading to G2/M arrest. Since T24 cells carry a mutated p53 gene, genistein-induced up-regulated p21 expression seem to cause of G2/M phase arrest of the cell cycle in regardless of p53 gene status. These results are consistent with several previous reports [27,28,33,38], indicating that genistein in T24 cells induces G2/M cell cycle arrest through a p53-independent mechanism.

Furthermore, based on the results of flow cytometry analysis and DAPI staining, we suggested that genistein-mediated apoptotic cell death was achieved with cell cycle arrest at the G2/M phase. In general, apoptosis can be divided into an extrinsic pathway initiated by the death receptor and an intrinsic pathway through the mitochondria [39,40]. The extrinsic pathway initiates when death ligands to their receptors, and then initiates the assembly and activation of caspase-8 that lead to activation of effector caspases, such as caspase-3, and -7 [39,41]. In contrast, the onset of the intrinsic pathway is accompanied by the release of cytochrome *c* from the mitochondria to the cytosol following increased mitochondrial permeability. The cytosolic release of cytochrome *c* activates effector caspases *via* the formation of apoptosome, which consists of cytochrome *c*, Apaf-1, and caspase-9. This pathway is precisely regulated by members of Bcl-2 family proteins [40,42,43]. Similar to some studies using other cancer cell lines [13,44,45], our results demonstrate that genistein activated caspase-8, -9 and -3, and induced PARP cleavage in T24 cells, which was associated the inhibition of the IAP family proteins that interfere with proteolytic activity by binding to caspases [46,47]. In addition, consistent with previous studies [13,14,48], mitochondrial dysfunction was induced in genistein-treated cells, as confirmed by the loss of MMP, which was accompanied by a down-regulation in the Bcl-2/Bax ratio and the cytosolic release of cytochrome *c*. However, genistein-induced growth reduction was significantly protected in the presence of a pan-caspase inhibitor. Therefore, based on those observations, we analogized that genistein-mediated apoptosis result from activation of the caspase-dependent pathway in T24 cells.

As is well known, induction of cell cycle arrest and apoptosis is tightly controlled by various cellular signaling pathways and regulatory molecules [39,49]. Among them, abnormal activation of the PI3K/Akt signal transduction pathway, a well-characterized cell growth signaling, is involved in the development of multiple human tumors, including bladder cancer [50,51,52]. Activated PI3K promotes activation of Akt, a downstream kinase of PI3K, which can inhibit apoptosis by protecting caspase cascade through phosphorylation of caspase-9, and enhances the expression of anti-apoptotic proteins [50,51]. Because these ultimately contribute to resistance to chemotherapy in cancer cells, PI3K and its regulatory factors are attractive targets for the therapy of cancers. Therefore, we examined whether this signaling pathway was associated with genistein-mediated cell cycle arrest and apoptosis in T24 cells, and found that the levels of the phosphorylated form of PI3K and Akt, but not the total levels, were suppressed in genistein-treated T24 cells. This means that the PI3K/Akt signaling pathway is inactivated by genistein treatment, and the results are similar to previous studies performed on several other cancer cell lines [13,14,48]. Furthermore, in line with a previous study using osteosarcoma cells [53], an inhibitor of PI3K, LY294002, significantly enhanced the apoptotic effect of genistein and further reduced cell viability, supposing that genistein-mediated apoptotic cell death is achieved by at least blocking the PI3K/Akt signaling pathway.

Accumulated evidence shows that various anti-cancer agents encourage suppression of cell proliferation for take-out of tumor cells through the promotion of oxidative properties [7,54]. According to the recent studies, it is announced that various bioactive compounds stimulated cell cycle arrest and apoptosis through ROS production, while inhibiting the PI3K/Akt signaling pathway [49,50]. These findings indicated that inactivation of the PI3K/Akt signaling pathway through increased ROS could be used for treatment strategy against cancer. Thus, we investigated whether genistein-stimulated growth arrest and apoptosis induction were associated with ROS generation and the PI3K/Akt signaling pathway. Consistent with previous studies [13,14,15,16,17], our findings presented that genistein significantly promoted ROS generation, while the scavenging of ROS markedly suppressed genistein-mediated disruption of MMP. These results indicated that ROS play as an upstream regulator to stimulate genistein-induced mitochondrial dysfunctions. In addition, our data confirmed that genistein-induced dephosphorylation of PI3K/Akt markedly inhibited under the presence of NAC. Subsequently, NAC pretreatment also effectively prevented genistein-mediated G2/M arrest and viability reduction, meaning that ROS may serve as a critical upstream regulator to the anti-cancer potentials of genistein.

Overall, our finding suggests that ROS production by genistein plays an important role in the induction of apoptosis associated with a cell cycle arrest in the G2/M phase in T24 cells. In addition, ROS acted as an upstream signal related to the effect of genistein on the blocking of the PI3K/Akt signaling pathway. However, further studies are needed to investigate the relationship between genistein-mediated inactivation of PI3K/Akt signaling pathway and other cellular signaling pathways, and the identification and role of intracellular organelles involved in ROS generation by genistein.

## 5. Conclusions

In this study, we demonstrated that genistein has anti-cancer effects *via* the mediation of apoptotic cell death associated with G2/M arrest of the cell cycle in human urinary bladder carcinoma T24 cells. Genistein-mediated cell cycle arrest was related to the down-regulation of G2/M regulatory cyclins, including cyclin A and cyclin B1, and up-regulation of Cdk inhibitor p21. In addition, genistein mediated apoptosis, due to the activation of caspases, which led to the degradation of PARP. Genistein also stimulated mitochondrial dysfunction, which was associated with a decrease in Bcl-2/Bax expression ratio, loss of MMPs, and cytochrome *c* release into cytosol. Furthermore, genistein inhibited the activity of the PI3K/Akt signaling pathway and induced excessive ROS production. Moreover, artificial interception of the PI3K/Akt signaling pathway improved genistein-mediated apoptotic cell death, and scavenging of intracellular ROS led to departing from G2/M cell cycle arrest. Based on these finding, we suggest that genistein has chemo-preventive potential by inducing G2/M arrest and apoptosis through ROS-dependent blocking of the PI3K/Akt signaling pathway in T24 cells.

## Supplementary Materials

The following are available online at https://www.mdpi.com/2076-3921/8/9/327/s1, Figure S1: Effect of genistein in C2C12 and V79-4 cells, Figure S2: Effect of genistein on apoptosis and cell cycle arrest in T24 cells for 24 h.
