# Supplementary material for: Induction of G2/M Cell Cycle Arrest and Apoptosis by Genistein in Human Bladder Cancer T24 Cells through Inhibition of the ROS-Dependent PI3k/Akt Signal Transduction Pathway
Source: Antioxidants (Basel). 2019 Aug 21;8(9):327. doi: 10.3390/antiox8090327 (PMC6769882; doi:10.3390/antiox8090327)
Supplement: Supplementary file 1 [file antioxidants-08-00327-s001.pdf]

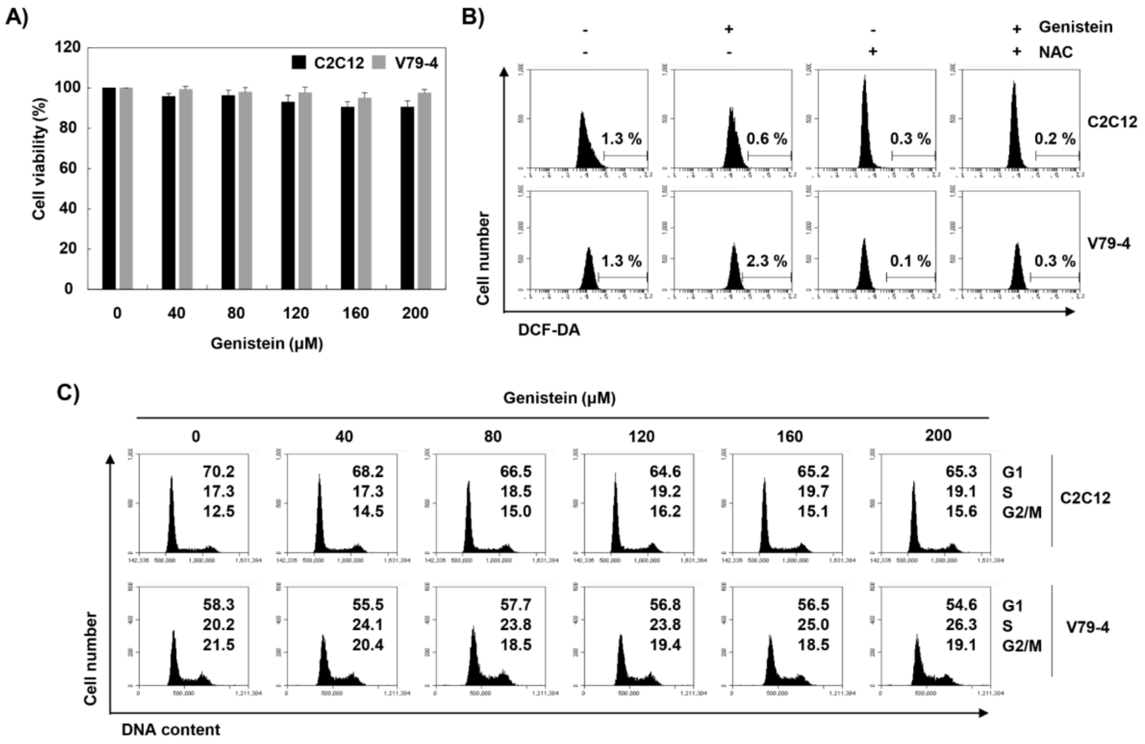

Figure 1. Effect of genistein in C2C12 cells and V79-4 cells.(**A**) Cells were treated with various concentration of genistein for 48 h. The cell viability was assessed by MTT assay. Each bar represents the mean ± SD of three independent experiments. (**B**) The cells were pre-treated with or without 10 mM N-acetyl-L-cysteine (NAC) for 1 h, before 160 μM genistein treatment for 1 h. The medium was discarded, and the cells were incubated for 20 min with new culture medium containing 5,6-carboxy-2′,7′-dichlorodihydrofluorescein diacetate (DCF-DA). ROS generation was measuredby flow cytometry. (**C**) Representative profiles. Quantification of the cell population (in percent) in different cell cycle phases of viable cells was shown.

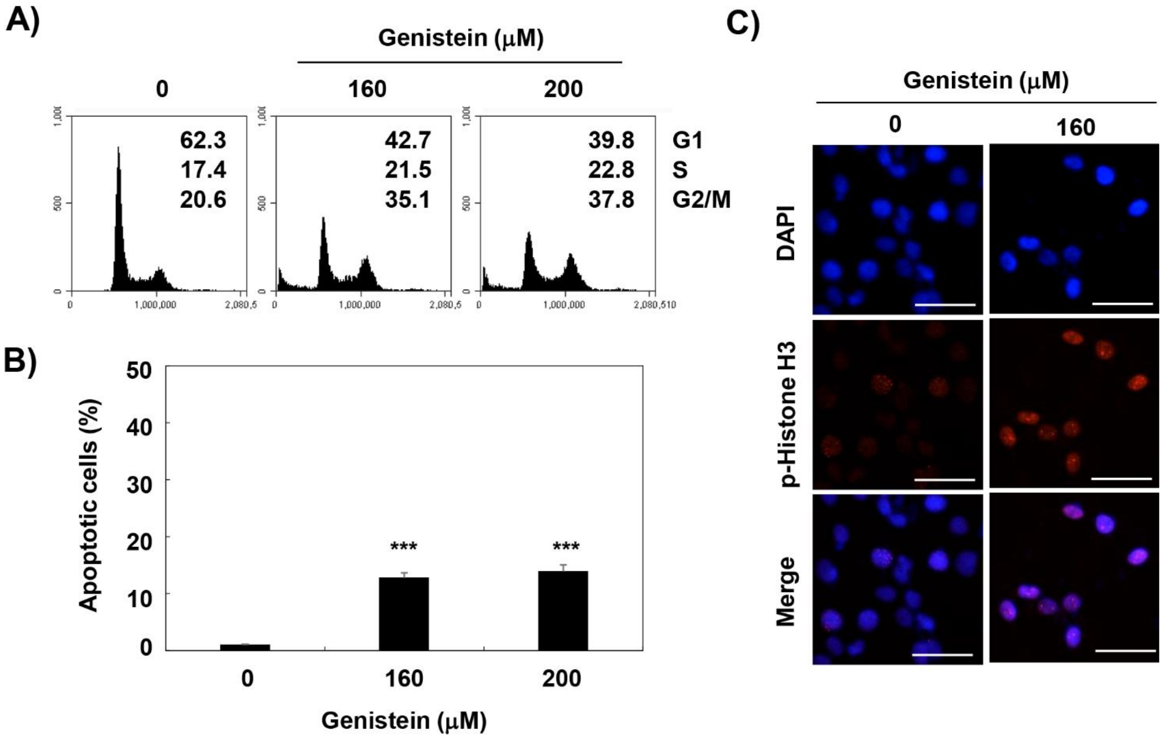

Figure 2. Effect of genistein on apoptosis and cell cycle arrest in T24 cells for 24 h.(**A**) Representative cell cycle profiles. Quantification of the cell population (in percent) in different cell cycle phases of viable cells was shown. (**B**) The cells were stained with annexin V-fluorescein isothiocyanate (FITC) and PI for flow cytometry analysis. The percentages of apoptotic cells were determined by expressing the numbers of Annexin V+ cells as percentages of all the present cells. The results are presented as the mean ± SD of three independent experiments (***$p<0.0001$ compared to control). (**C**) Cells were immuosteined with phospho-Histone H3 antibody (red) and DAPI (nuclear stain; blue), then the cells were observed under a fluorescence microscope (scale bar; 50 μm).
